# The impact of pneumococcal serotype replacement on the effectiveness of a national immunization program: a population-based active surveillance cohort study in New Zealand

**DOI:** 10.1016/j.lanwpc.2024.101082

**Published:** 2024-05-08

**Authors:** Andrew Anglemyer, Xiaoyun Ren, Charlotte Gilkison, Zoe Kumbaroff, Julie Morgan, Kara DuBray, Audrey Tiong, Arthur Reingold, Tony Walls

**Affiliations:** a Health Intelligence Team, Institute of Environmental Science and Research, Porirua, New Zealand; b Department of Preventive and Social Medicine, University of Otago, Dunedin, New Zealand; c Invasive Pathogens Laboratory, Institute of Environmental Science and Research, Porirua, New Zealand; d Department of Paediatrics, Dunedin Hospital, Dunedin, New Zealand; e Division of Epidemiology, School of Public Health, University of California, Berkeley, CA, USA; f Department of Paediatrics, University of Otago, Christchurch, New Zealand

**Keywords:** Pneumococcal disease, Serotype replacement, PCV, Antimicrobial resistance

## Abstract

**Background:**

In Aotearoa New Zealand (NZ) PCV7 was introduced in 2008, then PCV10 in 2011 and PCV13 in 2014. In 2017 PCV10 was re-introduced, replacing PCV13. In the present study, we investigate the resultant rapidly changing invasive pneumococcal disease (IPD) epidemiology.

**Methods:**

We compare the IPD incidence rate ratio (IRR) in NZ (2022 versus 2020) with other countries, and describe the IPD epidemiology (including trends in overall IPD and serotype 19A, and antimicrobial resistance) within NZ. Additionally, we performed a genomic-epidemiology investigation identifying the most common 19A sequence types and associated risk factors.

**Findings:**

Though IPD incidence rates have increased in the US and Australia (2021–22) after declines in 2020, in NZ the incidence rate is the highest since 2011 with a significantly higher IRR than US (p < 0.01). Incidence rates among children <2 and adults 65 or over in 2022 are the highest since 2009, driven by significant increases of serotype 19A (p = 0.01). Māori and Pacific peoples are experiencing the highest rates since 2009. Further, penicillin resistance among 19A isolates has increased from 39% (2012) to 84% (2021) (p = 0.02). Genomic sequencing identified the more virulent ST-2062 as most common among 19A isolates sequenced, increasing from 5% (2010) to 55% (2022).

**Interpretation:**

With very high incidence rates of IPD in NZ, inadequate protection against 19A, increasing resistance, and a more virulent 19A clade, targeted public health campaigns and increased PCV13 availability are needed.

**Funding:**

The NZ 10.13039/100009647Ministry of Health funds IPD surveillance and typing in NZ.

**Research in context**

**Evidence before this study**

Recent research has found an increase in invasive pneumococcal disease in recent years (2021–22) though the increases are varied between countries and appear to begin to occur in late 2021 or 2022. Additionally, we identified a few countries (Morocco 2012, Belgium 2017, New Zealand 2017, and El Salvador 2018) that switched from PCV13 to PCV10 in their Nation Immunization Programs (NIP). We found few comprehensive investigations of serotype 19A emerging as a replacement serotype, including genomic-epidemiology investigations of 19A sequence types, after switching from PCV13 to PCV10 in a NIP. This is likely due to the small number of countries that have shifted from PCV13 to PCV10. Indeed, El Salvador switched back to PCV13 2 years after PCV10 was re-introduced (2020), though serotyping since 2018 was not routinely done due to a lack of reagents so investigations into serotype trends were limited. Incidence of IPD due to serotype 19A has remained relatively low in Morocco before and after PCV13 was replaced with PCV10, though investigations into the impact of PCVs in Morocco have not typically differentiated between PCV10 and PCV13. In Belgium, researchers have previously reported significant increases in IPD due to 19A among children under 2 after switching from PCV13 to PCV10; their NIP switched back to PCV13 in 2020. Additionally, investigations in Belgium identified two predominant 19A clones, ST416 and ST994, that emerged after PCV10 was introduced.

**Added value of this study**

The current study addresses these absences in the literature by presenting evidence of an increase in invasive pneumococcal disease incidence in NZ which began at the onset of 2020, an increase that was earlier and greater than experienced in other countries. The relative burden of disease due to serotype 19A in NZ over the last 3 years is greater than observed globally previously, yielding the highest known invasive pneumococcal disease incidence rate for children <2 in the world in 2022. We provide strong evidence of 19A acting as a dominant replacement serotype after PCV13 was replaced with PCV10 in 2017. Further, we identified a unique genomic driver of 19A incidence in NZ--sequence type 2062, a sequence type not previously identified as very common in populations with surging 19A incidence rates.

**Implications of all the available evidence**

Active surveillance of invasive pneumococcal disease, particularly in countries with frequently changing NIPs, is vital in understanding the changing epidemiology. Further, the epidemiology of invasive pneumococcal disease in a country should be thoroughly considered before making substantial changes to NIPs. Researchers and policy makers should, when possible, utilize high-quality population-based surveillance data and analytical techniques to help inform decisions about changes to the immunization schedule and identify evidence of serotype replacement. These data can then not only help identify predictors of unique sequence types of dominant serotypes, but also help other countries make informed decisions with the best available data.

## Introduction

Though global vaccine programs have reduced mortality due to *Streptococcus pneumoniae*, invasive pneumococcal disease (IPD) is still responsible for nearly 300,000 deaths in children under 5 years annually.^1^ Some populations, particularly infants, elderly, and immunocompromised people are at higher risk for IPD, as well as specific ethnicities and indigenous populations.^2^

National immunization programs (NIPs) introduced the pneumococcal conjugate vaccine (PCV) in the early 2000s, initially covering 7 different serotypes (7-valent PCV7, Prevnar, Pfizer), followed by a 10-valent PCV (PCV10, Synflorix, GSK). A 13-valent PCV (PCV13, Prevnar 13, Pfizer) became available globally later; in mid-2023 a 20-valent PCV (Prevnar 20, Pfizer) became the latest PCV available for NIPs. Importantly, serotype 19A, included in PCV13 and PCV20 but not in PCV10, has emerged as a particularly dominant serotype, necessitating countries to re-evaluate their unique IPD epidemiology to ensure adequate coverage.^3^

In Aotearoa New Zealand (NZ) PCV7 was first introduced in 2008, followed by PCV10 in 2011 and PCV13 in 2014. However, in 2017 PCV10 was re-introduced in the NIP initially with a 3 + 1 schedule and then 2 + 1 from July 2020.^4^ We published our early concerns regarding increasing 19A incidence in 2020, in part because the percentage of PCV10-vaccine preventable cases that were 19A (assuming cross-protection from 19F in PCV10) increased from 45% to 80% since PCV10's re-introduction.^5^ Similar trends were found in other countries who used PCV10 in their NIP.^3^^,^^6^ In December 2022 the Pharmaceutical Management Agency, PHARMAC (the NZ Crown entity governing which vaccines are funded and on the vaccine schedule) announced a change back to PCV13 with a 2 + 1 schedule,^7^ but only for newborns without a catch-up immunization program.

In the first 2 years after the emergence of COVID-19 (2020–2021), a noted sustained decrease in IPD incidence was observed across 30 countries, though an increase in IPD has been observed in some of those countries toward the end of 2021.^8^ Australia and NZ had very early, strict public health measures against SARS-CoV2, in particular border closures from 2020–21,^9^ potentially making the countries more vulnerable to an immunity debt.^10^ However, with similar COVID-19 elimination strategies, but different NIPs (PCV10 in NZ and PCV13 in Australia), NZ has experienced a consistently, significantly higher incidence of IPD than Australia.^11^

In the present study, we aim to highlight the importance of an NIP to monitor for serotype replacement and changing epidemiology by describing the emerging IPD risks within NZ, with a focus on disproportionate risks between subgroups. Further, we aim to compare the recent overall IPD incidence rates between NZ and other countries. Additionally, we aim to describe the rapidly changing serotype diversity and investigate how 19A has emerged as a dominant replacement serotype in the absence of protection. Lastly, we aim to perform a genomic-epidemiology investigation identifying the most common 19A sequence types and their risk factors.

## Methods

### Study design and participants

In this population-based active surveillance cohort study, we assembled national-level IPD notification data reported from 2009 to 2022 to EpiSurv, the national notifiable disease database operated by the Institute of Environmental Science and Research (ESR); population estimates were collected from StatsNZ,^12^ New Zealand's official data agency. Additionally, we collated vaccine history among cases <5 years of age using the National Immunisation Register.^13^ All laboratory confirmed IPD cases in NZ since 2009 that met the case definition were included in this analysis. The case definition used by the NZ Ministry of Health states that IPD is confirmed with a detection of *Streptococcus pneumoniae* in a normally sterile site (e.g., meninges, cerebrospinal fluid, blood, pleural fluid, or joints); this would not include otitis media or sinusitis, for example. This study has ethics exemption as this analysis pertains to ongoing, active national-level surveillance activities of notifiable diseases within NZ. We followed the STROBE Statement in reporting our findings.

### Procedures

For comparison with NZ incidence rates, publicly available IPD count data were assembled from reported incidence counts from US^14^ and Australia,^15^ and after linking to population estimates^16^^,^^17^ we calculated incidence rates. Notified cases of IPD in NZ had serotyping performed at ESR's Invasive Pathogens Laboratory, and antimicrobial resistance (AMR) analyses were performed by ESR's Antibiotic Reference Laboratory. The EUCAST meningitis breakpoints were used to determine whether an isolate was resistant to penicillin.

Additionally, we performed whole genome sequencing (WGS) on a random selection of 19A isolates (Supplemental Table A1), selected by collection month, age group (<2, 2–4, 5–64, and 65+ years), and region (based on the submitting laboratory) from 2009 to 2022. Selection ensured that at least one isolate was selected for each combination. Additional details regarding methods for genome sequencing and analysis can be found in the Appendix. Genome assemblies were used to determine seven gene multi-locus sequence type (MLST)^18^ and penicillin-binding-protein type.^19^ Short-read sequencing data generated in this project are available at SRA, project number (PRJNA1100228). Assemblies available at PubMLST. Details of all genomes used in this study can be found in Supplemental Table A2.

### Statistical analysis

Annual incidence rates were calculated using the counts of cases that meet the case definition in a given calendar year divided by the census population estimates for that calendar year. To obtain exact 95% confidence intervals (CI) we assumed counts came from a Poisson distribution. Incidence rate ratios (IRR) were calculated comparing the incidence rates in 2022 (the last year PCV10 was used) versus 2017 (the year PCV10 was first re-introduced). To compare trends in IPD incidence since COVID-19 emergence (2020) between countries, we also calculated IRRs for each country comparing 2022 and 2020 incidence rates. We compared the IRRs between countries, and incidence rates between individual subgroups within a year, using tests for interaction.^20^

To evaluate the trends in serotype diversity, we employed two approaches: firstly, we evaluated the relative frequencies of PCV-specific serotypes over time; secondly, for each age group each year, we calculated Simpson's Diversity Index (SDI)^21^ and associated 95% CI.^22^ The SDI considers the relative richness and evenness of each serotype in a population and ranges from 0 (no diversity) to 1 (infinite diversity).^23^ Estimating SDI in children <2 and adults 65 years or older during various PCV eras can show the perturbation in pneumococcal populations, potentially resulting from changes in the NIPs. We evaluated the percentage of 19A isolates that were penicillin resistant and the percentage of all isolates that were 19A since 2012 with simple beta regression models.

To determine the odds of having any identified dominant sequence type of 19A among demographics, we performed a logistic regression. The covariates considered included age (<5, 5–49, 50–64, 65+ years), sex, prioritized ethnicity (European/Middle Eastern/Latin American/African, Māori, Pacific Peoples, Asian), and region (Northern Region/Auckland and Rest of NZ). A purposeful selection of covariates was used to develop initial multiple regression models.^24^ Full models were populated with all significant predictors (*p* < 0.1) from the univariate models and backwards elimination using Akaike's Information Criterion was used to help select the final model. We calculated adjusted odds ratios (a*OR*) and their respective 95% CI for each included covariate in the multivariable model. We compared the null model and preliminary models to the final model using likelihood ratio tests to determine if the residual deviance was significantly improved in the final model. All statistical analyses were performed using R (version 4.0.2, R Foundation for Statistical Computing, Vienna, Austria).

### Role of the funding source

This research received no specific funding, though IPD surveillance and typing in New Zealand is supported by funds from the New Zealand Ministry of Health. The Ministry of Health is not involved in the analysis, interpretation, design, or any aspect of this study. No author has been paid to write this manuscript.

## Results

### Trends in overall IPD incidence rate: NZ and other select countries

**Fig. 1:**
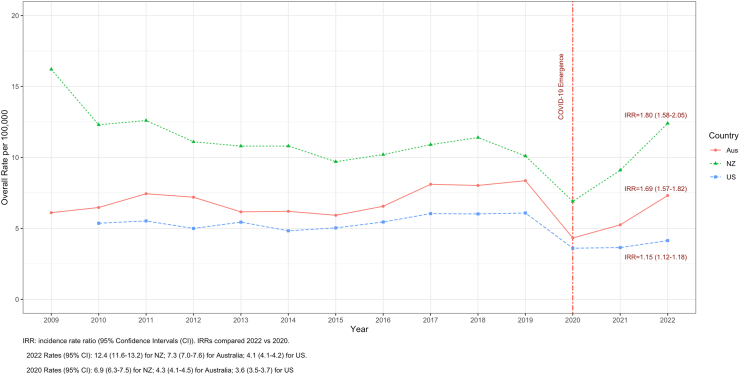
Overall IPD incidence rate per 100k for US, Australia, and NZ, 2009–22.

In 2022, the overall IPD incidence rate in Australia increased and is only about 10% lower than 2019 (pre-COVID emergence) (Fig. 1). Similarly, the overall IPD incidence rate in the US has increased, but is still about 30% lower than 2019. However, in NZ the IPD incidence rate in 2022 was 25% higher than 2019 and was the highest since 2011. The IRR comparing IPD incidence rates in 2022 to 2020 in NZ (IRR = 1.80; 95% CI 1.58–2.05) was significantly higher than the IRR for US (IRR = 1.15; 95% CI 1.12–1.18) (*p* < 0.01), though not significantly different than the IRR for Australia (IRR = 1.69; 95% 1.57–1.82) (*p* = 0.41).

### IPD epidemiologic trends in NZ

**Fig. 2:**
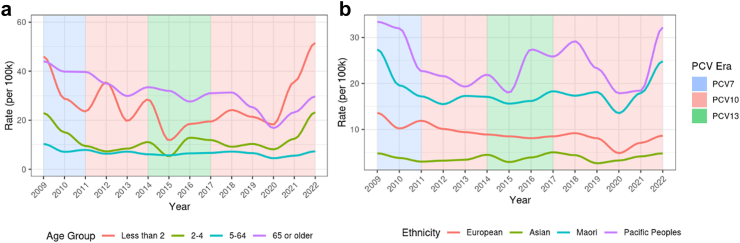
a and b: incidence rates of IPD by age group and ethnicity in NZ, 2009–2022.

The annual incidence rate of IPD was the highest among 65 years of age and over, followed by children <2 years of age from 2017 to 2019 (Fig. 2a and Supplemental Table A3). However, in 2020 the incidence rate among children <2 exceeded the rate for 65 years of age and over for the first time since 2009 (18.3 and 16.8 cases per 100,000, respectively). Further, the highest age-specific rates since 2017 have been among children <2 (35.7 and 51.5 cases per 100,000 in 2021 and 2022, respectively). Males have consistently had higher incidence rates of IPD than females since 2017, with significantly higher rates (13.8 cases per 100,000) than females (11.0 cases per 100,000) in 2022 (p < 0.01). Similarly, Pacific peoples have consistently had the highest incidence rates of IPD among all ethnic groups since 2017, reaching a high of 31.9 cases per 100,000 in 2022, significantly higher than all other ethnic groups in 2022 (p < 0.05 for all comparisons) (Fig. 2b and Supplemental Table A3). Māori also experienced their highest incidence rate since 2009 in 2022 (24.5 cases per 100,000).

### Rapidly changing epidemiology of IPD in NZ during PCV10 Re-introduction era, 2017–2022

The overall IPD incidence rate in 2022 (the last year of PCV10 in NIP) was significantly higher than in 2017 (the first year PCV10 was re-introduced in NIP) (IRR = 1.12; 95% CI 1.00–1.26) (Supplemental Table A4). Children under 2 and 2–4 year-olds experienced the biggest increases in IPD incidence rates, which were significantly higher when compared to 2017 (IRR = 2.63; 95% CI 1.65–4.33, and IRR = 1.95; 95% CI 1.18–3.32, respectively). Additionally, the IPD incidence rate among Māori in 2022 was significantly higher than in 2017 (IRR = 1.34; 95% CI 1.08–1.66), while incidence rates for European/Other and Asian ethnicity did not significantly change. Lastly, the IPD incidence rate among males in 2022 was significantly higher than in 2017 (IRR = 1.18; 95% CI 1.01–1.39).

### PCV switches in New Zealand's NIP and the effect on serotype replacement and distribution

**Fig. 3:**
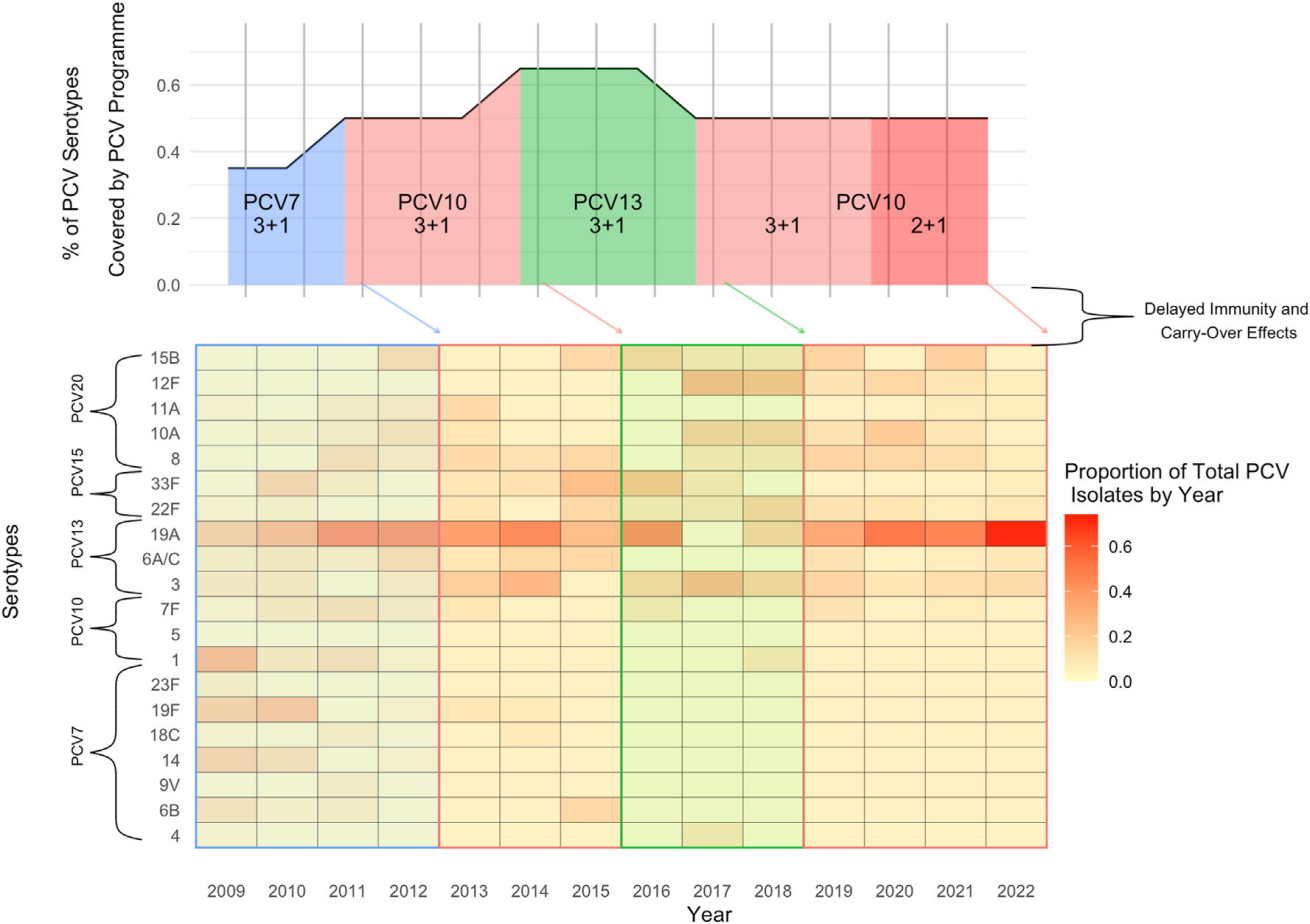
Changes in PCV programme, serotype coverage, and subsequent serotype distribution among children under 2 in New Zealand, 2009–2022.

The NIP in NZ has had multiple PCVs with varying coverage of vaccine-preventable serotypes (using PCV20, the licensed PCV with the broadest serotype coverage, as reference) since 2008 (Fig. 3). During the PCV13 era (2014–17), the percentage of vaccine-preventable serotypes covered by the NIP was the highest. As PCV10 was re-introduced as the primary PCV available in 2017 in the NIP, the universally common serotype 19A has emerged as a dominant replacement serotype in the absence of protection from the NIP. In fact, other than 19A, few IPD cases of PCV13-preventable serotypes have been observed among children <2 since 2017 (*n* = 24) (Fig. 3). Serotype 19A accounted for 33% of all IPD cases in 2022 among all ages, significantly increasing from 18% in 2014 (*p* = 0.03) (Supplemental Figure A1). Among children <5 since 2014, the proportion of IPD cases that were 19A has significantly increased from 31% to 59% in 2022 (*p* = 0.03) (Supplemental Figure A1 and Supplemental Table A5). Importantly, few IPD cases of PCV7-specific (*n* = 4) or PCV10-specific serotypes (*n* = 3) have been observed among children <5 since PCV10 was first re-introduced in 2017 (Supplemental Table A5), underscoring PCV10's direct effectiveness at prevention of IPD due to PCV10-specific serotypes.

### Effect of PCV changes on serotype diversity: contrasts between NZ and Australia

**Fig. 4:**
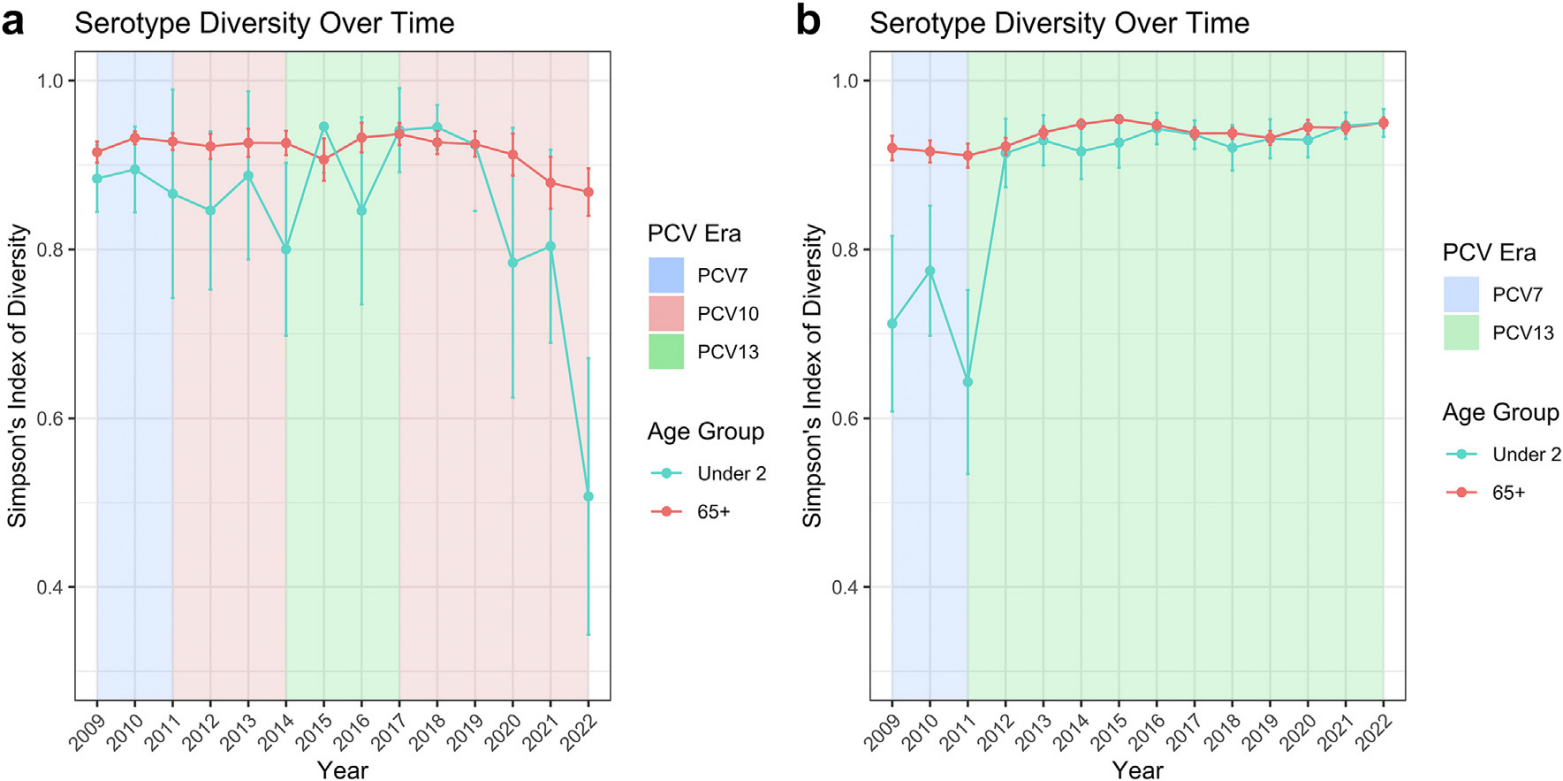
a and b: Simpson's diversity index in serotype distribution over time among children <2 and adults 65+ in New Zealand and Australia, 2009–2022. Note: Error bars displayed are 95% confidence intervals.

The SDI for adults in NZ had remained stable over time, though has decreased since 2017, reaching its lowest point in 2022 (0.88; 95% CI 0.83–0.93) since IPD became notifiable in 2008 (Fig. 4a). In contrast, though the SDI for adults in Australia has remained stable over time, it increased from approximately 0.92 at the end of the PCV7 era to approximately 0.95 in 2015–16 (Fig. 4b). Similarly, the SDI for children <2 in Australia increased rapidly from 0.65 in the last year of the PCV7 era (2011) to 0.90–0.95 from 2012 to 2022. The SDI for children <2 in NZ has been volatile over time due to the changing PCVs available at different times. The SDI was approximately 0.80–0.85 in the PCV13 era (2014–2017), and peaked in 2017–18 at approximately 0.92. Since 2018, the SDI has decreased, reaching a low in 2022 (0.48; 95% CI 0.30–0.62).

### IPD notifications among PCV-eligible children: immunization status and serotype 19A breakthrough cases

Since 2019, at least 85% of annual IPD cases in PCV-eligible children (born from January 1, 2008) had received at least one vaccination, though the PCV received was dependent on the PCV era in the NIP when they were vaccinated. From 2017 to 2022, the era of PCV10 re-introduction in the NIP, 145 PCV-eligible children were diagnosed with IPD due to 19A. Of these children, 5 (3.4%) were unvaccinated and 5 (3.4%) were breakthrough cases, vaccinated with PCV13 alone. In 2021, there were 75 PCV-eligible children who had been vaccinated and diagnosed with IPD. Of these 75, 52% (*n* = 39) of isolates would have been covered by PCV13 vaccine, of which 92% (*n* = 36) were 19A (*n* = 2 of the 36 were PCV13 breakthrough cases). In 2022, there were 99 PCV-eligible children who had been vaccinated and diagnosed with IPD. Of these 99, 61% (*n* = 60) were PCV13 vaccine preventable, of which 92% (*n* = 55) were 19A (no PCV13 breakthrough cases).

### Antimicrobial resistance and WGS of serotype 19A isolates in NZ

Penicillin resistance in invasive pneumococci has increased since 2012, driven by a significant increase in the prevalence of penicillin resistance among 19A IPD isolates (p = 0.02), increasing from 38.8% in 2012 (based on CLSI meningitis cut-off) to 84.1% in 2021 (based on EUCAST meningitis cut-off, Supplemental Figure A2). All but one of the penicillin-resistant serotype 19A isolates were also resistant to co-trimoxazole. Additional details on antimicrobial resistance among invasive pneumococci can be found in Supplemental Tables A6 and A7.

**Fig. 5:**
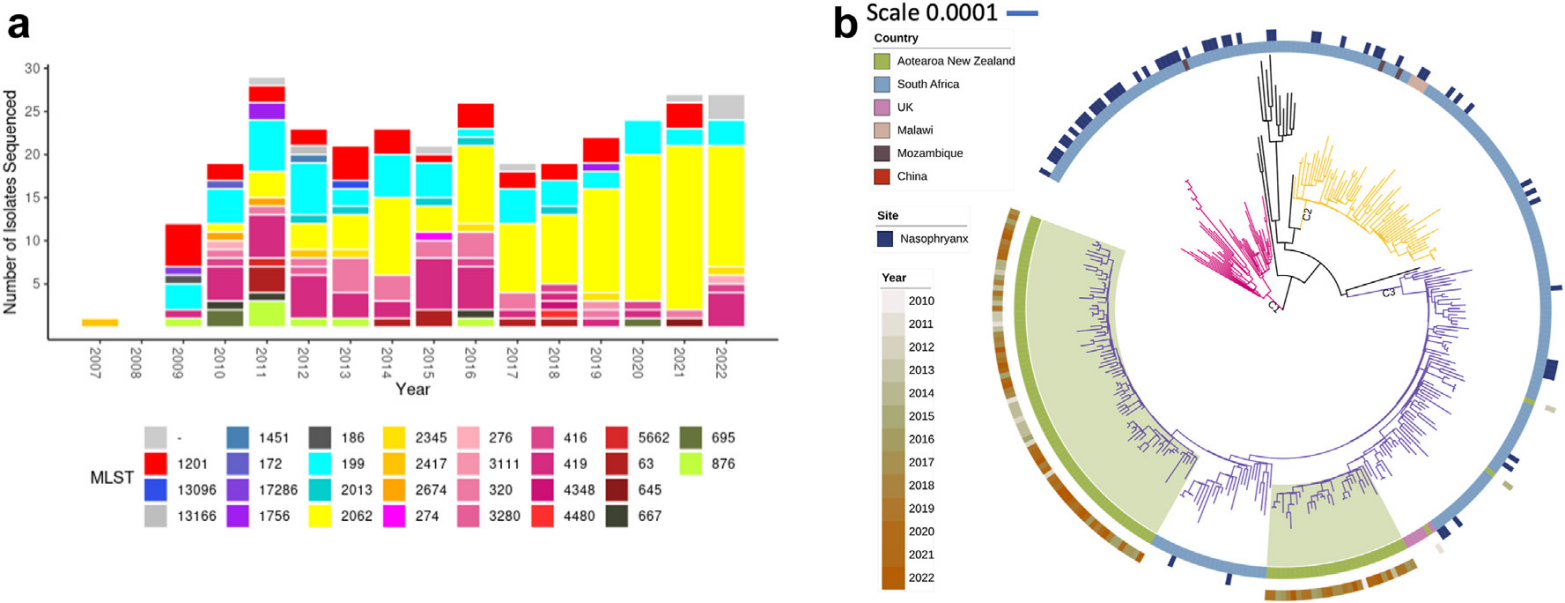
a and b Genomic sequencing of serotype 19A in Aotearoa New Zealand over time, 2007–2022. a) The number of different MLST in 19A isolates from 2007 to 2022 showing the predominance of ST-2062 (yellow) from 2017 to 2022. b) Phylogenetic analysis of NZ and international ST-2062 isolates. All NZ ST-2062 (Ring1, green) isolates can be divided into two subclades, highlighted in green, within C3. There are fewer carriage isolates (Ring2) within C3 when compared to C2 and C1. Both NZ sub-lineages within C3 are still circulating in NZ. There is no obvious clustering of isolates based on year collected (Ring3).

WGS analysis of 19A isolates found ST-2062 as the most common sequence type from 2016 to 2022. The relative percentage of ST-2062 among 19A isolates has increased from approximately 5% in sequenced 2010 isolates, to the most common sequence beginning in 2016 (Fig. 5a). Of the 110 NZ ST-2062 19A isolates sequenced, susceptibility data were available for 80 isolates, and all were resistant to penicillin (CLSI meningitis breakpoint before 2016, and EUCAST meningitis breakpoint after 2016). Nineteen of the sequenced isolates belonged to the highly resistant lineage ST-320,^25^ though only 7% of sequenced 2016–2022 isolates.

To understand how NZ ST-2062 isolates are related to international ST-2062 pneumococci, we constructed core SNP based maximum-likelihood phylogeny of NZ ST-2062 from this study and international ST-2062 genomes. Of the 233 international ST-2062 genomes available, 213 were from South Africa. We found the ST-2062 lineage can be divided into three well-supported (bootstrap value > 90) clades, C1 (49 genomes), C2 (65 genomes) and C3 (208 genomes, Fig. 5b) with all NZ isolates belonging to C3. Interestingly, of the 60 known carriage (marked as nasopharynx in PubMLST) derived isolates in this dataset, most were located in C1 (30/60), in contrast only 12/60 carriage isolates were located in C3. This may suggest the C3 lineage is possibly more likely to be associated with invasive disease (Fig. 5b). NZ ST-2062 isolates were grouped into two subclades within C3 suggesting there were at least two introduction events into NZ.

We found only age and geography, among covariates considered, to be significantly associated with having ST-2062 among 19A IPD cases (Supplemental Table A8). Specifically, in the final multivariable model, which had significantly improved residual deviance when compared to all other models, 19A cases living in the Northern region (including Northland and the Auckland area) were found to have significantly higher odds of having ST-2062 than 19A cases living in other regions in NZ, adjusting for age (*a*OR = 2.49; 95% CI 1.31–4.79); 19A cases 5 years of age and older all had significantly lower odds of having ST-2062 than children under 5, adjusting for region (5–49 years: *a*OR = 0.33; 95% CI 0.13–0.82, 50–64 years: *a*OR = 0.38; 95% CI 0.15–0.93, 65+ years: *a*OR = 0.25; 95% CI 0.11–0.54).

## Discussion

This surge in IPD in NZ, explained largely by 19A, underscores the impact an NIP that provides inadequate serotype coverage can have on disease burden. Importantly, these changes in risk could occur with any change to PCVs within an NIP. The epidemiology of IPD within a country at the time of the PCV change, coupled with the possibility that immunogenicity may be different with higher valency vaccines, may provide an environment for serotype replacement. Though serotype replacement may occur no matter what PCV vaccine is used, there is limited global evidence that 19A elicits cross-protection from PCV7 and PCV10^26^ and has proven itself persistent in terms of carriage and disease after PCV7 and PCV10 introduction.^27^ Dramatic increases in a predominant serotype in such a short amount of time are rarely observed in countries with a robust NIP, and may have been prevented with a PCV which includes coverage of 19A.

Though NIPs often result in vaccine serotypes declining due to herd immunity and vaccination, and non-vaccine serotypes grow due to serotype replacement, in NZ we have not observed an increase in any non-vaccine serotype except for 19A. The epidemiology of IPD in NZ has rapidly changed over the last 3–5 years, driven primarily by replacement serotype 19A, which has led to historically high incidence of IPD across all age groups. In fact, with an incidence rate of over 50 cases per 100,000 children <2 in 2022, we are unaware of any other high-income country with a higher burden of disease currently.

Serotype diversity has been unstable due to a NIP that has fluctuating coverage of serotypes in the community (5 changes in 12 years, increasing and decreasing serotype coverage and dosing), with the lowest diversity observed in 2022 for both <2 and 65 years or older in NZ. The SDI for children <2 in NZ in 2022 (0.48; 95% CI 0.30–0.62) is much lower than the lowest SDI for children in other high income countries since the early 2000s (∼0.70; 95% CI 0.65–0.75 in US in 2010).^23^ Low and decreasing SDI in children and older adults suggest few serotypes are causing most of the disease, due to the dominance of 19A. The indirect impact of the NIP on 65 years or older in NZ is especially important as there is no funded pneumococcal vaccine program in NZ for this age group. We note that the increase in IPD incidence rates in 2021–22 among 65 years or older in NZ was somewhat attenuated when compared to the rates in children <2, and these increases occurred after years of declining IPD rates from 2018 to 2020. We expect to continue to see increasing IPD incidence rates among 65 years or older in NZ from 2022 as indirect immunity effects of PCV10 in the younger population in previous years will be delayed.

The emergence of COVID-19 had an immediate impact on IPD incidence globally in 2020, though some countries reported increasing incidence by the end of 2021.^8^ In the present study we also observed an increase in IPD incidence rates in the US and Australia since 2020, though the increase in IPD incidence rates in NZ is unprecedented in the PCV era. In fact, while the overall IPD incidence rate decreased in 2020 in NZ, the decrease was attenuated in children <2 because the rate of 19A in this age group concurrently increased. Indeed, in the present study we found the overall IPD incidence rate in NZ in 2022 to be higher than in US and Australia. Importantly, as both Australia and NZ had very similar COVID-19 elimination and mitigation strategies from 2020 to 2021, the observed differences in IPD risk may be partially attributable to differing NIPs at the time. In fact, the IRR comparing IPD incidence rates in 2022 to 2020 in NZ for all ages was not significantly different than the IRR for Australia, suggesting the relative changes in IPD rates in 2022 compared to 2020 in both Australia and NZ were similar.

Further indication that the recent IPD experience in NZ is globally unique, typically rare severe clinical outcomes including *Streptococcus pneumoniae*-associated hemolytic uremic syndrome (spHUS) have increased.^28^ A recent analysis identified at least 19 IPD patients under 5 years of age with spHUS 2020–22 in NZ (all with 19A), while only nine cumulative cases were identified in the previous 20 years. Three-quarters were <2 years of age, and nearly 60% were Māori.

The overrepresentation of infectious diseases among indigenous and minority populations is a phenomenon that has been observed worldwide.^29^ However, differences in immunization rates alone cannot explain the higher relative increase of IPD among Māori and Pacific populations. More likely, the disproportionate effects of IPD are better accounted for by long standing inequities in socioeconomic determinants of health including, but not limited to housing conditions, and access to care.^30^ Addressing these inequities should remain a focus area of health policy and considered with any proposed changes to an NIP.

WGS analyses identified a growing proportion of 19A isolates that are ST-2062, accounting for 52% of 19A cases in 2022. This is important to note as ST-2062 is known to cause more illness among 19A carriers.^31^ Further, our phylogenetic analysis shows that all sequenced NZ ST-2062 isolates were located within a ST-2062 lineage that is maybe associated with invasive disease (Fig. 5b); within this lineage, NZ isolates are in two subclades suggesting clonal expansion within NZ following two importation events.

The association of NZ invasive isolates with C3 could be due to founders effect, without NZ carriage ST-2062 isolates we cannot determine if the NZ lineages are more invasive. Resistance among 19A isolates in NZ continues to grow, with nearly 90% of 19A isolates penicillin resistant–this may be due to the recent expansion of the ST-2062 strain in NZ. A rapid increase in 19A incidence was observed in Belgium after switching to PCV10 for 2 years, though their most common sequence type was ST-416 (47.6%), with less resistance and not observed among any of our sequenced 19A isolates.^32^ All NZ ST-2062 isolates sequenced were resistant to penicillin and have beta-lactam resistant alleles for penicillin-binding-protein (pbp) 1a, 2b and 2×.^33^ Other highly resistant lineages, such as ST-320, are present, but less common in NZ.

Though PCV13 for newborns has been re-introduced in NZ, carriage of 19A is likely still quite common in the community and IPD rates among older children and adults will likely remain elevated until PCV13 is made available to more children. In addition, immunization rates in NZ have been declining since 2015 with increasing disparities between Māori (range 86.6–91.6%)^34^ and Europeans. Therefore, the added impact of falling immunization rates on IPD incidence may not be fully realized in NZ yet. As PCV10 was part of the NIP through 2022, even if immunization rates were very high, there would likely be little impact on IPD incidence in children as very few IPD cases of PCV10-specific serotypes have been observed in children since 2017. Moreover, of 19A cases among PCV-eligible children diagnosed in 2022, 87% were vaccinated for age (all with PCV10 and none with PCV13 alone).

Our study has benefited from a surveillance system that captures all known IPD cases and identifies the serotypes of nearly all isolates. Further, the emergence of a dominant serotype 19A and the changing epidemiology in NZ offers compelling evidence for a stable immunization program that critically considers the changing epidemiology of IPD and the important role of WGS in understanding the sources of circulating, particularly virulent and penicillin-resistant *Streptococcus pneumoniae* strains. However, while our surveillance system was able to promptly identify increasing IPD incidence, we are unable to determine if there was a similar increase in non-invasive pneumococcal disease in the community. Additionally, another limitation of the present study is that the SDI does not account for changes to the schedule of PCV doses or changes in immunization rates.

## Contributors

A Anglemyer conceptualized and implemented the overall study design, implemented the epidemiologic methods, lead interpretation of analyses, and drafted the manuscript.

X Ren conceptualized and implemented the whole genome sequencing analysis, implemented the WGS methods, lead interpretation of the WGS analyses, and assisted in drafting the manuscript.

C Gilkison contributed to the conceptual and technical support early in the study design phase of the project, and provided epidemiologic and writing support on the manuscript.

Z Kumbaroff provided epidemiologic and writing support on the manuscript.

J Morgan provided technical expertise in laboratory methods and provided serotypes of isolates and writing support on the manuscript.

K DuBray provided clinical expertise in pediatric pneumococcal disease, interpretation of analyses, and writing support on the manuscript.

A Tiong was the overall technical laboratory lead and oversaw the serotyping and WGS methods, and provided support in writing of the manuscript.

A Reingold provided overall expertise and guidance in the implementation of an epidemiologic study of pneumococcal disease, and provided support in writing of the manuscript.

T Walls provided clinical expertise in pediatric pneumococcal disease, interpretation of analyses, and writing support on the manuscript.

## Data sharing statement

The aggregate data underlying the study is presented in the Supplementary material. Queries about data availability may be directed to: survqueries@esr.cri.nz.

## Declaration of interests

There are no declared conflicts of interest.
