# Supplementary material for: The impact of pneumococcal serotype replacement on the effectiveness of a national immunization program: a population-based active surveillance cohort study in New Zealand
Source: Lancet Reg Health West Pac. 2024 May 8;46:101082. doi: 10.1016/j.lanwpc.2024.101082 (PMC11091704; doi:10.1016/j.lanwpc.2024.101082)
Supplement: Appendix [file mmc1.docx]

# Appendix

#### *Methods for genome sequencing and analysis*

Pneumococcal isolates were grown on Columbia blood agar (CBA) plates (Fort Richard Laboratories, Auckland, New Zealand). DNA was extracted using the chemagic™360 instrument and sequenced using the Illumina NextSeq system using the 2x150bp paired-end format. Twenty-two 19A NZ genomes from the Global Pneumococcal Sequencing project (GPSP)^1^ were also included in our analysis. To put the NZ 19A isolates in the global genomic context, we compared NZ ST-2062 isolates to other ST-2062 genomes from the GPSP. Phylogenetic inference was performed using recombination-corrected core SNP alignment using iqtree2 v2.0.6.^2^ Core SNP alignment was generated with snippy v.4.6.0 using RefSeq genome NC_003098.1 as reference. Gubbins (Genealogies Unbiased By recomBinations In Nucleotide Sequences)^3^ as used to remove putative recombinant regions. Isolates sequenced in this project were assembled using the SKESA assembler v.2.3.0.^4^ Seven gene multi-locus sequence typing (MLST) was performed using mlst v. 2.19.0 using the PubMLST^5^ database (database version 11.10.2023).^6^ Penicillin-binding-protein typing was performed using amrfinder v3.11.4 (database version 2023-09-26.1)^7^ phylogenetic tree was visualised

using iTols^8^.

***Appendix References***

1. Gladstone RA, Lo SW, Lees JA, et al. International genomic definition of pneumococcal lineages, to contextualise disease, antibiotic resistance and vaccine impact. *EBioMedicine*. 2019;43:338-346. doi:10.1016/j.ebiom.2019.04.021

2. Minh BQ, Schmidt HA, Chernomor O, et al. IQ-TREE 2: New Models and Efficient Methods for Phylogenetic Inference in the Genomic Era. Teeling E, ed. *Mol Biol Evol*. 2020;37(5):1530-1534. doi:10.1093/molbev/msaa015

3. Croucher NJ, Page AJ, Connor TR, et al. Rapid phylogenetic analysis of large samples of recombinant bacterial whole genome sequences using Gubbins. *Nucleic Acids Res*. 2015;43(3):e15-e15. doi:10.1093/nar/gku1196

4. Souvorov A, Agarwala R, Lipman DJ. SKESA: strategic k-mer extension for scrupulous assemblies. *Genome Biol*. 2018;19(1):153. doi:10.1186/s13059-018-1540-z

5. Jolley KA, Bray JE, Maiden MCJ. Open-access bacterial population genomics: BIGSdb software, the PubMLST.org website and their applications. *Wellcome Open Res*. 2018;3:124. doi:10.12688/wellcomeopenres.14826.1

6. Seemann T. mlst Github. Published online 2023. https://github.com/tseemann/mlst

7. Feldgarden M, Brover V, Gonzalez-Escalona N, et al. AMRFinderPlus and the Reference Gene Catalog facilitate examination of the genomic links among antimicrobial resistance, stress response, and virulence. *Sci Rep*. 2021;11(1):12728. doi:10.1038/s41598-021-91456-0

8. Letunic I, Bork P. Interactive Tree Of Life (iTOL) v5: an online tool for phylogenetic tree display and annotation. *Nucleic Acids Res*. 2021;49(W1):W293-W296. doi:10.1093/nar/gkab301

9. European Committee on Antimicrobial Susceptibility Testing. *Breakpoint Tables for the Interpretation of MICS and Zone Diameters. Version 10.0*.; 2020.

***Appendix Tables***

*Table A1: Selection Scheme for Sequencing of Serotype 19A Isolates, 2009-22.*

|  | *2009-10*  *(n=31)* | *2011-12*  *(n=45)* | *2013-14*  *(n=33)* | *2015-16*  *(n=44)* | *2017-18*  *(n=38)* | *2019-20*  *(n=46)* | *2021-22*  *(n=54)* |
| --- | --- | --- | --- | --- | --- | --- | --- |
| *Age Group*  *<2*  *2-4*  *5-64*  *65+* | *9*  *4*  *9*  *9* | *5*  *8*  *14*  *18* | *4*  *7*  *12*  *10* | *5*  *8*  *17*  *14* | *1*  *7*  *13*  *17* | *8*  *5*  *16*  *17* | *14*  *9*  *15*  *16* |
| *Region*  *Far North*  *North*  *Middle*  *South/West* | *0*  *12*  *10*  *9* | *0*  *20*  *14*  *11* | *2*  *14*  *9*  *8* | *7*  *17*  *8*  *12* | *5*  *16*  *10*  *7* | *8*  *16*  *10*  *12* | *9*  *18*  *12*  *15* |
| *Quarter*  *1 (Jan-Mar)*  *2 (Apr-Jun)*  *3 (Jul-Sep)*  *4 (Oct-Dec)* | *8*  *8*  *11*  *4* | *8*  *16*  *17*  *4* | *5*  *8*  *15*  *5* | *6*  *16*  *16*  *6* | *6*  *8*  *17*  *7* | *6*  *5*  *16*  *19* | *8*  *25*  *17*  *4* |

*Table A2. Meta-data of all genomes used in this study. See attached excel spreadsheet.*

*Table A3: Characteristics of IPD Cases in NZ, 2017-2022*

|  | 2017 | | 2018 | | 2019 | | 2020 | | 2021 | | 2022 | |
| --- | --- | --- | --- | --- | --- | --- | --- | --- | --- | --- | --- | --- |
|  | n | rate^b^  (95% CI) | n | rate^b^  (95% CI) | n | rate^b^  (95% CI) | n | rate^b^  (95% CI) | n | rate^b^  (95% CI) | n | rate^b^  (95% CI) |
| Age  <2  2-4  5-64  65+ | 23  22  252  224 | 19·5  (12·4-29·2)  11·9  (7·5-18·0)  6·7  (5·9-7·6)  31·0  (27·1-35·4) | 29  17  277  234 | 24·2  (16·2-34·7)  9·2  (5·4-14·7)  7·2  (6·4-8·1)  31·3  (27·5-35·6) | 26  19  258  192 | 21·3  (13·9-31·3)  10·3  (6·2-16·1)  6·6  (5·8-7·4)  25·3  (21·8-29·1) | 22  15  180  133 | 18·3  (11·5-27·7)  8·1  (4·5-13·4)  4·5  (3·9-5·2)  16·8  (14·1-19·9) | 43  23  219  183 | 35·7  (25·9-48·1)  12·4  (7·9-18·7)  5·5  (4·8-6·3)  22·4  (19·3-25·9) | 62  43  292  235 | 51·5  (39·5-66·0)  23·2  (16·8-31·3)  6·1  (5·4-6·9)  28·0  (24·5-31·8) |
| Gender  Male  Female | 272  249 | 11·5  (10·2-13·0)  10·2  (9·0-11·6) | 277  280 | 11·5  (10·2-12·9)  11·3  (10·0-12·7) | 274  221 | 11·1  (9·8-12·5)  8·8  (7·7-10·1) | 178  172 | 7·1  (6·1-8·2)  6·7  (5·8-7·8) | 250  218 | 9·9  (8·7-11·2)  8·5  (7·4-9·7) | 348  284 | 13·7  (12·3-15·2)  11·0  (9·8-12·4) |
| Ethnicity  European/  Other  Māori  Pacific  Asian | 265  134  80  29 | 8·4  (7·4-9·4)  18·3  (15·3-21·7)  25·8  (20·5-32·2)  5·0  (3·4-7·2) | 292  129  92  26 | 9·0  (8·0-10·1)  17·3  (14·5-20·6)  29·2  (23·5-35·8)  4·4  (2·9-6·5) | 242  150  78  20 | 7·9  (6·9-9·0)  18·1  (15·3-21·3)  23·3  (18·4-29·0)  2·6  (1·6-4·1) | 148  114  61  25 | 4·7  (4-5·6·0)  13·5  (11·2-16·3)  17·9  (13·7-22·9)  3·2  (2·1-4·8) | 222  150  63  32 | 7·1  (6·2-8·1)  17·6  (14·9-20·7)  18·3  (14-23·4)  4·1  (2·8-5·8) | 264  209  110  37 | 8·4  (7·4-9·5)  24·5  (21·3-28·1)  31·9  (26·2-38·4)  4·8  (3·4-6·6) |
| Vaccination History^a^  3+ Doses  1-2 Doses  0 Doses | 42  6  11 | --  --  -- | 54  7  6 | --  --  -- | 48  5  9 | --  --  -- | 32  8  3 | --  --  -- | 54  21  9 | --  --  -- | 61  38  15 | --  --  -- |

- 1. Among PCV eligible cases. PCV became eligible in 2008 for all children born from January 1, 2008.
  2. Annual incidence rate per 100,000 (exact Poisson 95% confidence intervals)

*Table A4: Comparison of Invasive Pneumococcal Disease, 2017 (PCV10 Reintroduction Year) through 2022 (last year of PCV10 in NIP).*

|  | 2017 | 2022 | Incidence Rate Ratio Comparing Rates from 2022 to 2017 (95% CI) |
| --- | --- | --- | --- |
| Overall | 10·9 (521) | 12·4 (632) | 1·12 (1·00 to 1·26)* |
| Age  <2  2-4  5-64  65+ | 19·5 (23)  11·9 (22)  6·7 (252)  31·0 (224) | 51·5 (62)  23·2 (43)  6·1 (292)  28·0 (235) | 2·63 (1·65 to 4·33)*  1·95 (1·18 to 3·32)*  0·91 (0·77 to 1·07)  0·90 (0.75 to 1·08) |
| Sex  Males  Females | 11·5 (272)  10·2 (249) | 13·7 (348)  11·0 (284) | 1·18 (1·01 to 1·39)*  1·01 (0·86 to 1·20) |
| Ethnicity  European/Other  Asian  Māori  Pacific peoples | 8·4 (265)  5·0 (29)  18·3 (134)  25·8 (80) | 8·4 (264)  4·8 (37)  24·5 (209)  31·9 (110) | 1·00 (0·84 to 1·19)  0·95 (0·59 to 1·56)  1·34 (1·08 to 1·66)*  1·23 (0·93 to 1·65) |

*p value < 0·05

*Table A5: Number and percentage of invasive pneumococcal disease cases by serotype, serotypes covered by PCV7, PCV10 and PCV13, and age group, 2017-22*

|  | **2017** | | **2018** | | **2019** | | **2020** | | **2021** | | **2022** | |
| --- | --- | --- | --- | --- | --- | --- | --- | --- | --- | --- | --- | --- |
| **Sero-type** | **<5**  **years** | **≥5**  **years** | **<5**  **years** | **≥5**  **years** | **<5**  **years** | **≥5**  **years** | **<5**  **years** | **≥5**  **years** | **<5**  **years** | **≥5**  **years** | **<5**  **years** | **≥5**  **years** |
|  | **Cases (%)** | **Cases (%)** | **Cases (%)** | **Cases (%)** | **Cases (%)** | **Cases (%)** | **Cases (%)** | **Cases (%)** | **Cases (%)** | **Cases (%)** | **Cases (%)** | **Cases (%)** |
| 4 | 1 (2·2) | 18 (3·8) | 0 (0·0) | 4 (0·8) | 0 (0·0) | 8 (1·8) | 0 (0·0) | 3 (1·0) | 0 (0·0) | 3 (0·7) | 0 (0·0) | 2 (0·4) |
| 6B | 0 (0·0) | 1 (0·2) | 0 (0·0) | 2 (0·4) | 0 (0·0) | 4 (0·9) | 0 (0·0) | 1 (0·3) | 0 (0·0) | 1 (0·2) | 0 (0·0) | 3 (0·6) |
| 9V | 1 (2·2) | 3 (0·6) | 0 (0·0) | 3 (0·6) | 0 (0·0) | 1 (0·2) | 0 (0·0) | 0 (0·0) | 0 (0·0) | 0 (0·0) | 1 (1·0) | 0 (0·0) |
| 14 | 1 (2·2) | 1 (0·2) | 0 (0·0) | 2 (0·4) | 0 (0·0) | 3 (0·7) | 0 (0·0) | 1 (0·3) | 0 (0·0) | 2 (0·5) | 0 (0·0) | 0 (0·0) |
| 18C | 0 (0·0) | 1 (0·2) | 0 (0·0) | 1 (0·2) | 0 (0·0) | 0 (0·0) | 0 (0·0) | 0 (0·0) | 0 (0·0) | 2 (0·5) | 0 (0·0) | 1 (0·2) |
| 19F | 0 (0·0) | 14 (2·9) | 0 (0·0) | 6 (1·2) | 0 (0·0) | 9 (2·0) | 0 (0·0) | 5 (1·6) | 0 (0·0) | 10 (2·5) | 0 (0·0) | 6 (1·1) |
| 23F | 0 (0·0) | 1 (0·2) | 0 (0·0) | 2 (0·4) | 0 (0·0) | 2 (0·4) | 0 (0·0) | 1 (0·3) | 0 (0·0) | 0 (0·0) | 0 (0·0) | 0 (0·0) |
| **PCV7** | **3 (6·7)** | **39 (8·2)** | **0 (0·0)** | **20 (4·0)** | **0 (0·0)** | **27 (6·0)** | **0 (0·0)** | **11 (3·5)** | **0 (0·0)** | **18 (4·5)** | **1 (1·0)** | **12 (2·2)** |
| 1 | 0 (0·0) | 2 (0·4) | 1 (2·2) | 4 (0·8) | 0 (0·0) | 2 (0·4) | 0 (0·0) | 1 (0·3) | 0 (0·0) | 0 (0·0) | 0 (0·0) | 0 (0·0) |
| 5 | 0 (0·0) | 0 (0·0) | 0 (0·0) | 0 (0·0) | 0 (0·0) | 0 (0·0) | 0 (0·0) | 0 (0·0) | 0 (0·0) | 0 (0·0) | 0 (0·0) | 0 (0·0) |
| 7F | 0 (0·0) | 30 (6·3) | 0 (0·0) | 27 (5·3) | 1 (2·2) | 8 (1·8) | 0 (0·0) | 6 (1·9) | 1 (1·5) | 6 (1·5) | 0 (0·0) | 10 (1·9) |
| **PCV10** | **0 (0·0)** | **32 (6·7)** | **1 (2·2)** | **31 (6·1)** | **1 (2·2)** | **10 (2·2)** | **0 (0·0)** | **7 (2·2)** | **1 (1·5)** | **6 (1·5)** | **0 (0·0** | **10 (1·9)** |
| 3 | 4 (8·9) | 29 (6·1) | 2 (4·3) | 31 (6·1) | 2 (4·4) | 26 (5·8) | 2 (5·4) | 23 (7·3) | 3 (4·5) | 18 (4·5) | 6 (5·8) | 41 (7·8) |
| 6A | 0 (0·0) | 2 (0·4) | 0 (0·0) | 3 (0·6) | 0 (0·0) | 1 (0·2) | 0 (0·0) | 0 (0·0) | 0 (0·0) | 0 (0·0) | 0 (0·0) | 0 (0·0) |
| 19A^a^ | 4 (8·9) | 56 (11·8) | 4 (8·7) | 71 (13·9) | 10 (22·2) | 55 (12·2) | 18 (48·6) | 53 (16·9) | 32 (48·5) | 106 (26·4) | 62 (59·6) | 145 (27·5) |
| **PCV13** | **8 (17·8)** | **87 (18·3)** | **6 (13·0)** | **105 (20·6)** | **12 (26·7)** | **82 (18·2)** | **20 (54·1)** | **76 (24·3)** | **35 (53·0)** | **124 (30·9)** | **68 (65·4)** | **186 (35·3)** |
| 6C | 1 (2·2) | 11 (2·3) | 1 (2·2) | 13 (2·5) | 2 (4·4) | 9 (2) | 1 (2·7) | 5 (1·6) | 3 (4·5) | 15 (3·7) | 4 (3·8) | 17 (3·2) |
| 6D | 0 (0·0) | 0 (0·0) | 0 (0·0) | 0 (0·0) | 0 (0·0) | 0 (0·0) | 0 (0·0) | 0 (0·0) | 0 (0·0) | 1 (0·2) | 0 (0·0) | 0 (0·0) |
| 7B | 0 (0·0) | 0 (0·0) | 0 (0·0) | 0 (0·0) | 0 (0·0) | 0 (0·0) | 0 (0·0) | 0 (0·0) | 0 (0·0) | 0 (0·0) | 0 (0·0) | 1 (0·2) |
| 7C | 0 (0·0) | 3 (0·6) | 0 (0·0) | 2 (0·4) | 0 (0·0) | 2 (0·4) | 1 (2·7) | 4 (1·3) | 0 (0·0) | 3 (0·7) | 0 (0·0) | 5 (0·9) |
| 8 | 1 (2·2) | 48 (10·1) | 1 (2·2) | 47 (9·2) | 2 (4·4) | 62 (13·8) | 2 (5·4) | 56 (17·9) | 3 (4·5) | 75 (18·7) | 2 (1·9) | 140 (26·6) |
| 9N | 0 (0·0) | 12 (2·5) | 0 (0·0) | 22 (4·3) | 1 (2·2) | 20 (4·4) | 0 (0·0) | 6 (1·9) | 1 (1·5) | 7 (1·7) | 1 (1·0) | 9 (1·7) |
| 10A | 2 (4·4) | 9 (1·9) | 2 (4·3) | 14 (2·7) | 1 (2·2) | 5 (1·1) | 3 (8·1) | 3 (1·0) | 3 (4·5) | 5 (1·2) | 0 (0·0) | 3 (0·6) |
| 10F | 0 (0·0) | 1 (0·2) | 0 (0·0) | 0 (0·0) | 0 (0·0) | 0 (0·0) | 0 (0·0) | 0 (0·0) | 0 (0·0) | 0 (0·0) | 0 (0·0) | 0 (0·0) |
| 11A | 0 (0·0) | 20 (4·2) | 0 (0·0) | 12 (2·3) | 0 (0·0) | 12 (2·7) | 0 (0·0) | 10 (3·2) | 1 (1·5) | 13 (3·2) | 1 (1·0) | 12 (2·3) |
| 12F | 3 (6·7) | 19 (4) | 5 (10·9) | 47 (9·2) | 2 (4·4) | 37 (8·2) | 3 (8·1) | 28 (8·9) | 2 (3·0) | 12 (3) | 1 (1·0) | 6 (1·1) |
| 13 | 0 (0·0) | 1 (0·2) | 0 (0·0) | 0 (0·0) | 0 (0·0) | 3 (0·7) | 0 (0·0) | 3 (1·0) | 1 (1·5) | 2 (0·5) | 0 (0·0) | 0 (0·0) |
| 15A | 2 (4·4) | 18 (3·8) | 5 (10·9) | 12 (2·3) | 0 (0·0) | 10 (2·2) | 0 (0·0) | 10 (3·2) | 0 (0·0) | 2 (0·5) | 0 (0·0) | 2 (0·4) |
| 15B | 1 (2·2) | 14 (2·9) | 2 (4·3) | 13 (2·5) | 2 (4·4) | 10 (2·2) | 0 (0·0) | 8 (2·6) | 6 (9·1) | 11 (2·7) | 0 (0·0) | 10 (1·9) |
| 15C | 2 (4·4) | 3 (0·6) | 0 (0·0) | 1 (0·2) | 0 (0·0) | 1 (0·2) | 0 (0·0) | 1 (0·3) | 0 (0·0) | 0 (0) | 0 (0·0) | 0 (0) |
| 16F | 0 (0·0) | 15 (3·2) | 0 (0·0) | 14 (2·7) | 1 (2·2) | 10 (2·2) | 0 (0·0) | 11 (3·5) | 1 (1·5) | 18 (4·5) | 0 (0·0) | 11 (2·1) |
| 17F | 1 (2·2) | 3 (0·6) | 0 (0·0) | 9 (1·8) | 1 (2·2) | 5 (1·1) | 0 (0·0) | 2 (0·6) | 0 (0·0) | 2 (0·5) | 0 (0·0) | 3 (0·6) |
| 18A | 0 (0·0) | 3 (0·6) | 0 (0·0) | 0 (0·0) | 0 (0·0) | 0 (0·0) | 0 (0·0) | 0 (0·0) | 0 (0·0) | 0 (0·0) | 0 (0·0) | 3 (0·6) |
| 18F | 0 (0·0) | 0 (0·0) | 0 (0·0) | 1 (0·2) | 0 (0·0) | 0 (0·0) | 0 (0·0) | 1 (0·3) | 0 (0·0) | 0 (0·0) | 0 (0·0) | 0 (0·0) |
| 20 | 0 (0·0) | 1 (0·2) | 0 (0·0) | 1 (0·2) | 0 (0·0) | 3 (0·7) | 0 (0·0) | 0 (0) | 0 (0·0) | 2 (0·5) | 0 (0·0) | 1 (0·2) |
| 21 | 0 (0·0) | 3 (0·6) | 1 (2·2) | 1 (0·2) | 3 (6·7) | 3 (0·7) | 0 (0·0) | 1 (0·3) | 2 (3·0) | 2 (0·5) | 0 (0·0) | 0 (0) |
| 22A | 0 (0·0) | 1 (0·2) | 1 (2·2) | 1 (0·2) | 0 (0·0) | 0 (0) | 0 (0·0) | 0 (0) | 0 (0·0) | 0 (0) | 0 (0·0) | 0 (0) |
| 22F | 4 (8·9) | 31 (6·5) | 2 (4·3) | 47 (9·2) | 3 (6·7) | 50 (11·1) | 1 (2·7) | 14 (4·5) | 1 (1·5) | 25 (6·2) | 3 (2·9) | 26 (4·9) |
| 23A | 1 (2·2) | 11 (2·3) | 3 (6·5) | 13 (2·5) | 1 (2·2) | 14 (3·1) | 0 (0·0) | 11 (3·5) | 0 (0·0) | 7 (1·7) | 0 (0·0) | 9 (1·7) |
| 23B | 1 (2·2) | 16 (3·4) | 3 (6·5) | 20 (3·9) | 5 (11·1) | 14 (3·1) | 3 (8·1) | 12 (3·8) | 1 (1·5) | 16 (4) | 4 (3·8) | 23 (4·4) |
| 29 | 0 (0·0) | 0 (0·0) | 0 (0·0) | 0 (0·0) | 0 (0·0) | 0 (0·0) | 0 (0·0) | 0 (0·0) | 0 (0·0) | 0 (0·0) | 0 (0·0) | 2 (0·4) |
| 31 | 0 (0·0) | 7 (1·5) | 0 (0·0) | 5 (1) | 0 (0·0) | 6 (1·3) | 0 (0·0) | 2 (0·6) | 0 (0·0) | 1 (0·2) | 0 (0·0) | 3 (0·6) |
| 33F | 2 (4·4) | 14 (2·9) | 1 (2·2) | 19 (3·7) | 0 (0·0) | 7 (1·6) | 0 (0·0) | 7 (2·2) | 0 (0·0) | 6 (1·5) | 1 (1·0) | 8 (1·5) |
| 34 | 0 (0·0) | 1 (0·2) | 2 (4·3) | 2 (0·4) | 0 (0·0) | 5 (1·1) | 0 (0·0) | 1 (0·3) | 0 (0·0) | 3 (0·7) | 0 (0·0) | 2 (0·4) |
| 35B | 0 (0·0) | 5 (1·1) | 0 (0·0) | 2 (0·4) | 0 (0·0) | 4 (0·9) | 0 (0·0) | 3 (1) | 0 (0·0) | 6 (1·5) | 1 (1·0) | 6 (1·1) |
| 35F | 0 (0·0) | 5 (1·1) | 0 (0·0) | 3 (0·6) | 0 (0·0) | 1 (0·2) | 0 (0·0) | 2 (0·6) | 0 (0·0) | 2 (0·5) | 0 (0·0) | 2 (0·4) |
| 37 | 0 (0·0) | 0 (0) | 0 (0·0) | 1 (0·2) | 0 (0·0) | 0 (0) | 0 (0·0) | 2 (0·6) | 0 (0·0) | 1 (0·2) | 0 (0·0) | 0 (0) |
| 38 | 2 (4·4) | 12 (2·5) | 2 (4·3) | 3 (0·6) | 0 (0·0) | 1 (0·2) | 1 (2·7) | 0 (0) | 0 (0·0) | 1 (0·2) | 0 (0·0) | 0 (0) |
| 42 | 0 (0·0) | 1 (0·2) | 0 (0·0) | 0 (0) | 0 (0·0) | 0 (0) | 0 (0·0) | 0 (0) | 0 (0·0) | 0 (0) | 0 (0·0) | 0 (0) |
| Other^b^ | 0 (0·0) | 2 (0·4) | 1 (2·2) | 3 (0·6) | 1 (2·2) | 10 (2·2) | 1 (2·7) | 2 (0·6) | 0 (0·0) | 2 (0·5) | 0 (0·0) | 1 (0·2) |
| **Non-PCV** | **23 (51·1)** | **290 (60·9)** | **32 (69·6)** | **328 (64·2)** | **25 (55·6)** | **304 (67·6)** | **16 (43·2)** | **205 (65·5)** | **25 (37·9)** | **240 (59·7)** | **18 (17·3)** | **305 (57·9)** |
| **Not tested** | 11 (24·4) | 28 (5·9) | 7 (15·2) | 27 (5·3) | 7 (15·6) | 27 (6·0) | 1 (2·7) | 14 (4·5) | 5 (7·6) | 14 (3·5) | 17 (16·3) | 14 (2·7) |
| **Total^c^** | **45 (100)** | **476 (100·0)** | **46 (100)** | **511 (100)** | **45 (100)** | **450 (100)** | **37 (100)** | **313 (100)** | **66 (100)** | **402 (100)** | **104 (100)** | **527 (100)** |

^a^ PCV10 (Synflorix®) has some unknown level of cross-reactivity to serotype 19A, one of the three additional serotypes included in PCV13 (Prevenar13®).

^b^ Includes non-typeable serotypes.

^c^ Total number of isolates from culture-positive cases referred to ESR for serotyping for each age group, including those that were not able to be tested.

*Table A6. Antimicrobial susceptibility among isolates from invasive pneumococcal disease cases, 2021*

|  | EUCAST clinical breakpoints^a^ | | | | Susceptibility (%) | | |
| --- | --- | --- | --- | --- | --- | --- | --- |
|  | S^b^ | I^b^ | | R^b^ | S^b^ | I^b^ | R^b^ |
| Antibiotic | Minimum inhibitory concentration (MIC, mg/L) | | | |  | | |
| Penicillin | | | | | | | |
| Meningitis | <=0·06 | - | >=0·12 | | 64·3 | - | 35·7 |
| non-meningitis^c^ | <=0·06 | 0·12-2 | >=4 | | 64·3 | 35·2 | 0·4 |
| Cefotaxime | | | | | | | |
| Meningitis | <=0·5 | - | >=1 | | 100·0 | - | 1·3 |
| non-meningitis | <=0·5 | 1-2 | >=4 | | 98·7 | 1·3 | 0·0 |
|  | Zone diameter (mm) | | | |  |  |  |
| Chloramphenicol | >=21 | - | <=20 | | 99·6 | - | 0·4 |
| Clindamycin^d^ | >=19 | - | <=18 | | 97·4 | - | 2·6 |
| Co-trimoxazole | >=13 | 10-12 | <=9 | | 68·7 | 0·4 | 30·8 |
| Erythromycin | >=22 | 19-21 | <=18 | | 95·2 | 0·4 | 4·4 |
| Moxifloxacin | >=22 | - | <=21 | | 100 | - | 0·0 |
| Rifampicin | >=22 | 17-21 | <=16 | | 100 | 0·0 | 0·0 |
| Tetracycline | >=25 | 22-24 | <=21 | | 95·2 | 0·4 | 4·4 |
| Vancomycin | >=16 | - | <=15 | | 100 | - | 0·0 |

a European Committee on Antimicrobial Susceptibility Testing ^9^.

b S=susceptible standard dosing (high likelihood therapeutic success using standard dosing). I=susceptible increased exposure (likelihood of therapeutic success with adjustments to dosing or concentrations), and R=resistant.

c EUCAST also provide several additional dose-specific penicillin breakpoints for pneumonia. Based on the susceptible breakpoint (MIC<=0·5) for a dose of 1·2 g 6 hourly 96·0% of isolates would be categorised as susceptible.

d The percentage resistant given is for constitutive clindamycin resistance. No isolates had inducible clindamycin resistance.

*Table A7. Penicillin and cefotaxime resistance among isolates*

*from invasive pneumococcal disease cases, 2021*

| Age group (years) | Penicillin | | Cefotaxime | |
| --- | --- | --- | --- | --- |
|  | Resistant^a^  MIC ≥0·12 mg/L | | Resistant  MIC ≥1 mg/L | |
|  | Number | %^b^ | Number | %^b^ |
| <2  (n=20) | 7 | 35·0 | 1 | 5·0 |
| 2-4  (n=10) | 8 | 80·0 | 0 | 0·0 |
| 5-64 (n=106) | 29 | 27·4 | 0 | 0·0 |
| 65+  (n=91) | 37 | 40·7 | 2 | 2·2 |
| All ages (n= 227) | 81 | 35·7 | 3 | 1·3 |

a EUCAST meningitis breakpoints; no susceptible increased exposure^9^.

b Percentage of the isolates from the cases within the age group.

*Table A8. Associations between Demographic Characteristics and Carriage of ST-2062 among Serotype 19A IPD Patients, 2015-2022*

|  | Univariate Analyses | Multivariable Analyses^1^ |
| --- | --- | --- |
|  | *u*OR (95% CI) | *a*OR (95% CI) |
| Age  <5  5-49  50-64  65+ | Ref  0·35 (0·14-0·83)  0·38 (0·15-0·93)  0·26 (0·11-0·55) | Ref  0·33 (0·13-0·82)  0·38 (0·15-0·93)  0·25 (0·11-0·54) |
| Ethnicity  European/MELAA  Māori  Pacific Peoples  Asian | Ref  3·47 (0·87-17·72)  1·33 (0·27-7·58)  0·40 (0·05-2·42) | <dropped> |
| Sex  Female  Male | Ref  1·52 (0·85-2·73) | <dropped> |
| Region  Rest of New Zealand  Auckland/Northern Region | Ref  2·42 (1·31-4·54) | Ref  2·49 (1·31-4·79) |

1.Likelihood ratio tests comparing the multivariable model to all other combinations suggest improved residual deviance in the final model (*p* < 0·001)

*u*OR=unadjusted odds ratio; *a*OR=adjusted odds ratio

***Appendix Figures***

*Figure A1: Proportion of IPD Isolates That Were Serotype 19A, by PCV Era and Age Group**


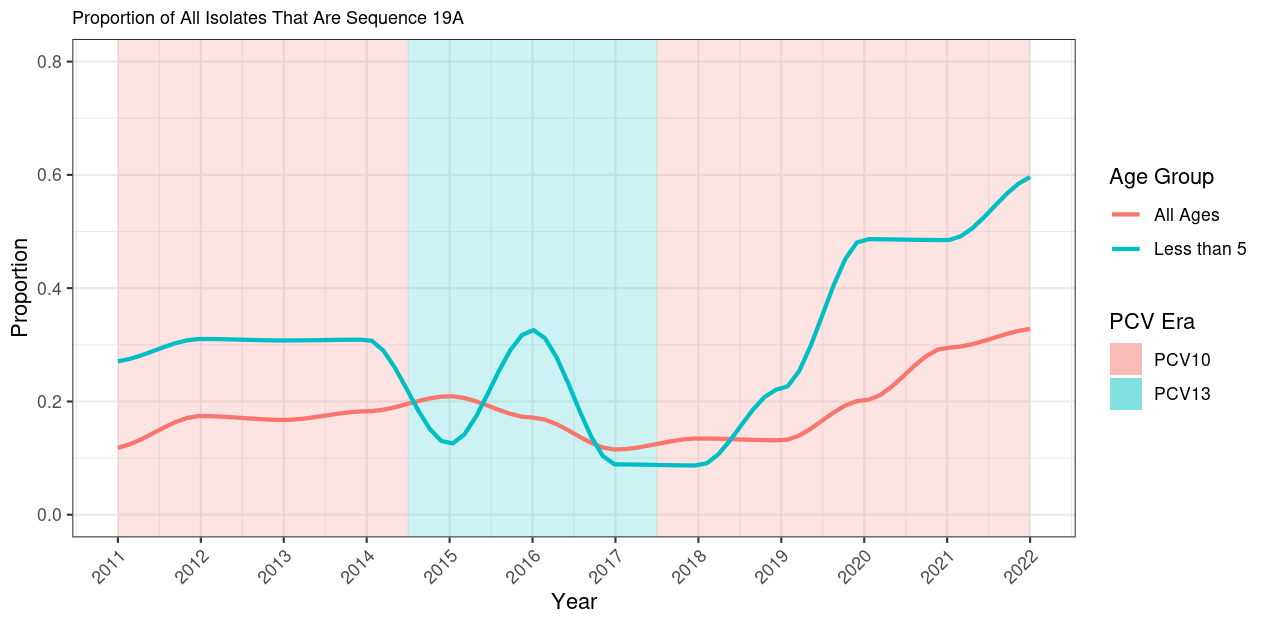


*Linear model of proportions that are 19A from 2017 to 2022 was significant (*p*<0·01 for children <5 and *p*=0·01 for all).

Penicillin-resistant, serotype 19A isolates were susceptible to all other antibiotics tested except for one isolate that was also erythromycin resistant and two isolates that were both erythromycin and tetracycline resistant. In 2021 the next most prevalent serotype among the penicillin-resistant isolates was type 35B, which accounted for 6·2% of penicillin-resistant isolates. All serotype 8 isolates were fully susceptible to the antimicrobials tested.

*Figure A2: Rates of Penicillin-Resistance among pneumococci from invasive disease cases, 2012-2021**


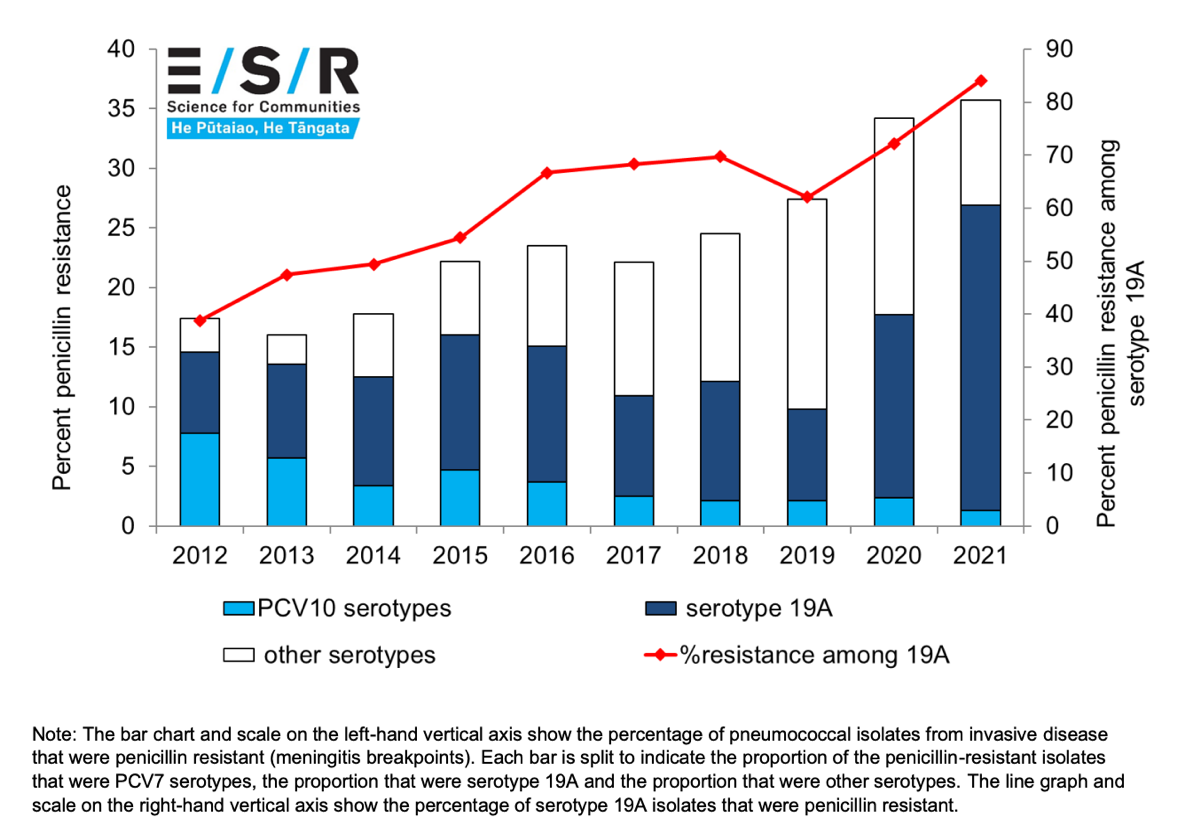


There was a change to EUCAST susceptibility testing methods in 2016, which means that not all susceptibility testing

results from 2016 to 2021 are directly comparable. However, the rates of penicillin resistance (based on the CLSI before

2016 and EUCAST after 2016 meningitis resistance breakpoint of MIC ≥0·12 mg/L) are comparable and therefore trends

in these rates of resistance for the 2012–2021 period are presented

**p* value < 0·01
